# Genetic manipulation for abiotic stress resistance traits in crops

**DOI:** 10.3389/fpls.2022.1011985

**Published:** 2022-09-21

**Authors:** Nardana Esmaeili, Guoxin Shen, Hong Zhang

**Affiliations:** ^1^ Department of Basic and Translational Sciences, School of Dental Medicine, University of Pennsylvania, Philadelphia, PA, United States; ^2^ Zhejiang Academy of Agricultural Sciences, Sericultural Research Institute, Hangzhou, China; ^3^ Department of Biological Sciences, Texas Tech University, Lubbock, TX, United States

**Keywords:** abiotic stresses, crop production, drought stress, heat stress, salinity stress, transgenic plants

## Abstract

Abiotic stresses are major limiting factors that pose severe threats to agricultural production. Conventional breeding has significantly improved crop productivity in the last century, but traditional breeding has reached its maximum capacity due to the multigenic nature of abiotic stresses. Alternatively, biotechnological approaches could provide new opportunities for producing crops that can adapt to the fast-changing environment and still produce high yields under severe environmental stress conditions. Many stress-related genes have been identified and manipulated to generate stress-tolerant plants in the past decades, which could lead to further increase in food production in most countries of the world. This review focuses on the recent progress in using transgenic technology and gene editing technology to improve abiotic stress tolerance in plants, and highlights the potential of using genetic engineering to secure food and fiber supply in a world with an increasing population yet decreasing land and water availability for food production and fast-changing climate that will be largely hostile to agriculture.

## Introduction

About 40 years ago, Boyer wrote that the negative impacts of environmental factors could reduce crop yield by around 70%, which would be a disaster for this planet (Boyer, 1982). He also proposed exploring crops’ genetic potential to improve their yields. The adverse effects of abiotic stresses such as heat, drought, and salinity that are accelerated by climate change and global warming are becoming serious threats to today’s world (Esmaeili et al., 2019). It is projected that by mid-21st century the global temperature will increase by around 4 °C above 20th century, which will pose a great risk to global food security (IPCC, 2014). According to a new report from United Nations the world population is expected to reach 9.8 billion by 2050 and 11.2 billion by 2100 (World population prospects: the 2017 revision, UN Department of Economic and Social Affairs). To feed the growing population on earth, an increase of 44 million metric tons of grains per year is required (Tester and Langridge, 2010). Reports show that about 10% of arable land can be classified as stress-free zones, indicating that crops growing in the remaining 90% of arable land are facing some types of environmental stresses (Dita et al., 2006), with some severely reducing agricultural productivity annually. Many crop improvement strategies such as conventional breeding, tissue culture, chemical priming, and genetic engineering have been deployed to overcome the threats posed by abiotic stresses (Kumar et al., 2020; Rivero et al., 2022).

Plants are subjected to various stresses due to their sessile nature. Thus, plants have evolved several strategies and elaborate mechanisms to perceive, respond, and adapt to adverse environments. Plant response to unfavorable environments is manifested by triggering molecular networks, including signal transduction, up-regulation of stress-related genes, and production of proteins and metabolites that help plants to handle adverse conditions. In many cases, plants show a similar response to different environmental stresses. For instance, plant responses to salt, drought, and cold stresses share similar genes that are triggered by these stresses (Chinnusamy et al., 2007). The polygenic nature of plant response to abiotic stresses makes plant improvement very difficult (Dita et al., 2006). Despite numerous efforts in studying the underlying mechanism of plant response to environmental stresses, the plant stress response is still not adequately understood (Reguera et al., 2012). In recent years the “omics” approach (e.g., proteomics, genomics, and metabolomics) assisted scientists in unraveling the signaling pathways that regulate plant response to stresses, which could result in a large gain in crops productivity (Van Emon, 2016). Using model plants such as *Arabidopsis thaliana* provided a fundamental platform in the plant biotechnology arena. Since most of these studies aiming at improving plant stress tolerance were conducted with the model plants and remained in the model systems, a very limited number of transgenic crops were created and tested in the field, and only a few transgenic crops were released commercially (Umezawa et al., 2006). Most studies with model plants were conducted in the lab under artificial conditions mimicking the environment, but plants growing in the field respond to complex environmental conditions, which vary in time, duration, and intensity. Therefore, it is crucial to focus on different aspects of combined stresses to successfully develop crops that can withstand multiple stresses in the field (Mittler, 2006). The advantages of ‘stacking’ or ‘pyramiding’ of stress-related genes in crops offer a great potential to prepare futural crops for a fast-changing environment (Esmaeili et al., 2019; Wijewardene et al., 2020; Esmaeili et al., 2021; Balasubramaniam et al., 2022).

Although conventional breeding has improved crop yield considerably, it was not very successful in enhancing abiotic stress tolerance in crops (Hu and Xiong, 2014; Kumar et al., 2020; Rivero et al., 2022). This lack of success is partially due to breeders’ preference to test their genetic materials under optimum conditions. The complex nature of abiotic stresses and variability in plants’ sensitivity to different stresses during their life cycle further complicate the selection criteria for increased stress tolerance in conventional breeding. Therefore, it is imperative to adopt an alternative approach that could be employed to improve abiotic stress tolerance and enhance crop yield and quality. Genetic engineering is an alternative strategy to generate transgenic crops that can withstand the fast-changing environment. In the last two decades, transgenic plants have been generated by altering the expression of different genes responsible for a specific trait *via* different transformation methods (Ashraf and Akram, 2009). According to the International Service for the Acquisition of Agro-Biotech Applications (ISAAA, 2019), the United States, Brazil, Argentina, Canada, and India are the top five countries that grow genetically engineered crops. The major transgenic crops adopted by GMO-growing countries in 2019 were soybean, maize, cotton, and canola (ISAAA, 2019). In this review, we overview recent advances in developing transgenic plants with improved abiotic stress tolerance and discuss the roadmap for further enhancing abiotic stress tolerance in crops.

## Manipulation of single genes in plants *via* transgenic approach

The in-depth study of plant stress response involves a coalition of pathways in terms of signal perception and transduction cascades, activation of transcription factors, expression or modulation of stress-related genes, production of various functional proteins and enzymes, production of osmolytes, increased antioxidation capacities against reactive oxygen species (ROS), and alterations to biochemical, physiological, and cellular aspects of plant cellular metabolisms. In this section, we will discuss the employment of the transgenic approach in the alteration of single gene expression, which improves plant performance under major abiotic stress conditions.

### Engineering plants for enhanced salt tolerance

Since around 25% of the earth’s outermost layer is affected by salt, salinity is considered a growing agricultural problem threatening global food security (Rao et al., 2006; Abdelraheem et al., 2019). Soil salinization is caused by both human and natural processes. Accumulation of salt in soil inhibits water uptake by roots, disrupts ion homeostasis, and inversely affects plant growth and development (Zhu, 2001). To combat the adverse effects of salinity, plants have developed different strategies, including salt exclusion and compartmentalization into vacuoles. The Na^+^ accumulation in vacuoles and export out of cells are controlled by the activities of proton pumps and antiporters operating at the tonoplast and the plasma membrane (Gaxiola et al., 2001; Shi et al., 2003). The Na^+^/H^+^ antiporter exchanges vacuolar H^+^ for cytosolic Na^+^ and thus sequesters Na^+^ ions into vacuoles (Apse et al., 1999) or exports Na^+^ ions out of cells, while the proton gradient force generated by vacuolar membrane-bound H^+^-pyrophosphatase (V-PPase) and ATPase and plasma membrane-bound ATPase contribute to these processes (Gaxiola et al., 2001; Zhu, 2001).

Studies have shown that overexpression of several stress-related genes to reduce the uptake of toxic ions such as Na^+^ in the cytosol could improve plant salt tolerance (**Table 1**). Since the first report by Gaxiola et al. (2001), researchers have overexpressed the *vacuolar H^+^-pyrophosphatase* (*V-PPase*) genes in different crops such as alfalfa (Bao et al., 2009), rice (Kim et al., 2020), sugarcane (Kumar et al., 2014), cotton (Lv et al., 2008; Pasapula et al., 2011), peanut (Qin et al., 2013), wheat (Lv et al., 2015), and tobacco (Gao et al., 2006) to improve the salt tolerance and yield in those crops. The *V-PPase*-overexpressing plants also produce more robust root systems, which is due to more active auxin polar transport, thereby leading to more efficient water and nutrient absorption (Li et al., 2005). Extensive research has also been conducted on overexpression of the plant *vacuolar Na^+^/H^+^ antiporter* gene, *NHX*, to improve salt tolerance in transgenic plants. For instance, transgenic tomato plants overexpressing the *Arabidopsis vacuolar Na^+^/H^+^ antiporter gene 1* (i.e., *AtNHX1*) could grow, flower, and produce fruits under 200 mM NaCl. At the same time, high Na^+^ and Cl^-^ contents were detected in leaves, but not in the fruits (Zhang and Blumwald, 2001). Overexpression of *AtNHX1* in cotton also improved salt tolerance, increased photosynthetic rate, and enhanced fiber production in transgenic lines after salt treatment (He et al., 2005). Transgenic canola, wheat, and maize plants overexpressing *AtNHX1* demonstrated improved salt tolerance and produced more biomass and grain yield under saline conditions (Zhang et al., 2001; Xue et al., 2004; Yin et al., 2004). Furthermore, overexpression of *AtNHX1* orthologs such as *AtNHX5*, *OsNHX1*, *MdNHX1*, *TaNHX2*, *PgNHX1*, and *LeNHX2* also improved salt tolerance in crops including rice, eggplant, soybean, apple, and tomato. (Fukuda et al., 2004; Chen et al., 2007a; Verma et al., 2007; Li et al., 2010; Cao et al., 2011; Li et al., 2011a; Yarra et al., 2012; Huertas et al., 2013; Yarra and Kirti, 2019).

**Table 1:** Improving plant salt stress tolerance through genetic engineering.

Gene	Gene source	Transgenic host	Improved traits	Reference
*OVP1*	*Oryza sativa*	Rice	Enhanced salt tolerance, membrane stability, and higher chlorophyll content	Kim et al., 2020
*OsNHX1*			Enhanced salt tolerance	Fukuda et al., 2004; Chen et al., 2007a
*OsDREB1B*			Enhanced salt and drought tolerance,	Datta et al., 2012
*OsDREB1F*			Improved salt, drought, and cold tolerance	Wang et al., 2008
*OsMYB6*			Increased salt and drought tolerance, higher proline content, higher CAT and SOD activities	Tang et al., 2019
*SNAC2*			Increased salt and cold stress, higher germination and growth rate under salt	Hu et al., 2008
*P5CS*	*Vigna aconitifolia*		Improved salt tolerance, better root growth, and biomass development	Anoop and Gupta, 2003
*AtNHX5*	*Arabidopsis thaliana*		Enhanced salt and drought tolerance, dry weight, and chlorophyll content; Reduced membrane damage	Li et al., 2011a
*PgNHX1*	*Pennisetum glaucum*		Improved salt tolerance, robust root	Verma et al., 2007
*AVP1*	*Arabidopsis thaliana*	Alfalfa	Improved salt and drought tolerance, and photosynthetic rate	Bao et al., 2009
*TsVP*	*Thellungiella halophila*	Cotton	Improved salt tolerance, shoot and root growth, and photosynthetic performance, reduced MDA and membrane leakage	Lv et al., 2008
*AVP1*	*Arabidopaia thaliana*		Improved salt and drought tolerance, increased fiber production	Pasapula et al., 2011
*AtNHX1*			Salt tolerance, photosynthetic rate, fiber production	He et al., 2005
*SeVP*	*Salicornia europaea*	Wheat	Enhanced salt tolerance and nitrogen deficiency	Lv et al., 2015
*P5CS*	*Vigna aconitifolia*		Enhanced salt tolerance, high proline content	Sawahel and Hassan, 2002
*AtNHX1*	*Arabidopsis thaliana*		Improved salt tolerance, biomass, higher grain yields and heavier and larger grains	Xue et al., 2004
*AtNHX1*	*Arabidopsis thaliana*	Tomato	Improved salt tolerance, low Na^+^ and high K^+^ contents in fruit	Zhang and Blumwald, 2001
*TaNHX2*	*Triticum aestivum*		Increased salt tolerance, RWC, and germination rate	Yarra et al., 2012
*LeNHX2*	*Lycopersicum esculenyum*		Enhanced salt tolerance and higher K^+^ uptake	Huertas et al., 2013
*SlSOS2*	*Solanum lycopersicum*		Enhanced salt tolerance, earlier flowering, and higher fruit production	Huertas et al., 2012
*AtNHX1*	*Arabidopsis thaliana*	Canola	Improved salt tolerance up to 200 mM NaCl, Seed yield and seed oil quality were not affected by salt stress	Zhang et al., 2001
*TaNHX2*	*Triticum aestivum*	Eggplant	Improved salt tolerance, growth, higher RWC and chlorophyll content, reduced MDA and ROS	Yarra and Kirti, 2019
*MdNHX1*	*Malus × domestica* Borkh	Apple	Improved salt tolerance, high K^+^/Na^+^ ratio in the leaves	Li et al., 2010
*GmCLC1*	*Glycine max*	Soybean	Enhanced salt tolerance, lower relative electrolyte leakage	Wei et al., 2016
*GmDREB6*			Improved salt tolerance and high proline content	Nguyen et al., 2019
*GsCLC-c2*	*Glycine soja*		Improved salt tolerance, Cl^−^ and NO_3_^−^ homeostasis	Wei et al., 2019
*OsDREB2A*	*Oryza sativa*		Improved salt tolerance, higher soluble sugars and free proline accumulation	Zhang et al., 2013
*P5CS*	*Solanum torvum*		Improved salt tolerance, leaf area, relative chlorophyll content, and number of fresh pods	Zhang et al., 2015
*TaNHX2*	*Triticum aestivum*		Improved salt tolerance and increased flowers	Cao et al., 2011
*ZmbZIP4*	*Zea mays*	Maize	Enhanced salt, drought, and osmotic stress tolerance	Ma et al., 2018
*AtNHX1*	*Arabidopsis thaliana*		Enhanced salt tolerance and germination rate	Yin et al., 2004
*P5Cs*	*Arabidopsis thaliana*	Potato	Increased salt tolerance and proline content, less altered tuber yield and weight	Hmida-Sayari et al., 2005
*SOS*	*Ipomoea batatas*		Improved salt tolerance, high SOD activity and proline content; Reduced MDA content	Gao et al., 2012
*AVP1*	*Arabidopsis thaliana*	Peanut	Improved salt and drought tolerance, biomass, photosynthetic rate, and higher yields	Qin et al., 2013
*AVP1*	*Arabidopsis thaliana*	sugarcane	Enhanced salt and drought stresses, and robust root system	Kumar et al., 2014

The genes in the *salt overly sensitive* (*SOS*) signaling pathway, *SOS1, SOS2*, and *SOS3*, play a substantial role in salt tolerance in plants by excluding Na^+^ ions at the cellular level and maintaining ion homeostasis in root cells (Shi et al., 2000). Overexpression of *SOS2* that encodes a calcineurin-interacting protein kinase from *Solanum lycopersicum* in tomato increased salt tolerance (Huertas et al., 2012). In a study by Yue et al. (2012), transgenic tobacco plants overexpressing the plasma membrane N^+^/H^+^ antiporter gene *SOS1* showed enhanced salt tolerance, and they grew much better than wild-type plants when irrigated with 150 mM NaCl. A similar result was obtained by Gao et al. (2012) when *SOS* genes were overexpressed in sweet potato plants. Compartmentalization of Cl^-^ ions in vacuole using chloride channel proteins (CLCs) is another mechanism to counter salt stress in plants to decrease Cl^-^ levels in the cytosol, thereby maintaining ion homeostasis in plant cells. Overexpression of *chloride channel protein* genes *GmCLC1* and *GsCLC-c2* in soybean improved salt stress tolerance in transgenic plants (Wei et al., 2016; Wei et al., 2019), while silencing *GhCLCg*-1 in upland cotton compromised salt stress tolerance, indicating the importance of CLCs in plant response to salt stress (Liu et al., 2021).

Plant response to environmental stresses is regulated by a series of stress-related genes that are modulated by specific transcription factors. The functions of some transcription factors, e.g., MYC, bZIP, WRKY, NAC, and AP2, in salt signaling pathways were identified (Golldack et al., 2011). Overexpression of the *dehydration responsive element binding protein* genes, *DREB1B* and *OsDREB1F* in rice (Wang et al., 2008; Datta et al., 2012), *GmDREB2* in tobacco (Chen et al., 2007b), *GmDREB6* and *OsDREB2A* in soybean (Zhang et al., 2013; Nguyen et al., 2019), and *DREB1A* in potato (Behnam et al., 2006) improved salt stress tolerance. The transgenic rice overexpressing the *stress-responsive NAC transcription factor* gene *SNAC2* showed improved salinity tolerance (Hu et al., 2008). The myeloblastosis oncogene encoded proteins (MYBs) are another group of transcription factors whose function in abiotic stress response in plants is well understood. Recently, Tang et al. (2019) showed that overexpression of the rice gene *OsMYB6* improves salinity and drought stress tolerance in transgenic rice. *OsMYB6*-overexpressing rice plants also produced a larger amount of proline, and they showed increased catalase (CAT) and superoxide dismutase (SOD) activities (Tang et al., 2019). Also, overexpression of *ZmbZIP4* in maize improved salt and drought tolerance at the seedling stage and enhanced osmotic stress adjustments (Ma et al., 2018).

Osmotic adjustment is a vital plant response to abiotic stresses. The accumulation of osmolytes under abiotic stress conditions is well documented. These compatible osmolytes include amino acids and their derivatives (e.g., proline and glycine betaine), soluble sugars (e.g., trehalose and mannitol), and sugar alcohols (Suprasanna et al., 2016). Several reports showed that manipulating genes that control the production of low molecular weight metabolites such as proline (an essential amino acid in plants) improves plant tolerance to abiotic stresses, including salinity and drought. Transgenic soybean overexpressing the proline biosynthetic gene *P5CS* that encodes the Δ′-pyrroline-5-carboxylate synthetase (P5CS) demonstrated increased salt tolerance with higher proline content (Zhang et al., 2015). This is consistent with previous results from transgenic wheat, potato, and indica rice plants overexpressing *P5CS* (Sawahel and Hassan, 2002; Anoop and Gupta, 2003; Hmida-Sayari et al., 2005). Glycine betaine (GB) is an essential osmolyte that protects plants against osmotic stress by stabilizing membrane and photosynthetic machinery under salt, drought, and cold stresses. Transgenic rice was developed by overexpressing the *choline oxidase A* gene, *codA*, in chloroplast and cytosol. In both cases, higher production of GB and improved salt stress tolerance were observed. Since rice does not produce GB, expression of *codA* in transgenic rice is essential for the increased abiotic stress tolerance in rice (Mohanty et al., 2002).

Plants have developed mechanisms such as antioxidant molecules and enzymes to respond to the overproduction of reactive oxygen species (ROS) generated under abiotic stress conditions (Grene, 2002; Devireddy et al., 2021). The antioxidant molecules such as ascorbate and glutathione can interact directly with ROS and therefore reduce ROS content in plant cells. The antioxidant enzymes such as superoxide dismutase (SOD), catalase (CAT), ascorbate peroxidase (APX), and glutathione reductase (GR) can scavenge ROS efficiently in plant cells (Anjum et al., 2016). Numerous studies on overexpression of antioxidant genes to improve plant tolerance to abiotic stresses were reported (Gill and Tuteja, 2010). For instance, overexpression of *Cu/Zn SOD* and *APX* in potatoes increased salt stress tolerance (Yan et al., 2016).

### Improved drought tolerance in transgenic crops

Drought is a major stress that results in huge crop loss. Around one-third of the lands on earth are located in arid and semiarid regions where drought severely limits agricultural production (Abdelraheem et al., 2019). Therefore, developing crops that can grow and produce high yields under water deficit conditions is urgently needed. Plant response to drought stress is very complex which involves different pathways, and the intensity and span of drought stress alter both their interaction and individual responses. In the past few decades, many studies have focused on generating transgenic crops with improved drought tolerance (**Table 2**). The first commercialized drought-tolerant corn known as DroughtGard, MON87460, which expresses the cold shock protein B gene from *Bacillus subtilis*, was developed by Monsanto (Monsanto Co., St. Louis, MO, USA) and released in 2009 (Nemali et al., 2014; Liang, 2016a). However, its success in the market is not very clear.

**Table 2:** Improving drought stress tolerance in transgenic crops.

Gene	Gene source	Transgenic host	Improved traits	Reference
*VaNCED1*	*Vitis amurensis*	Grapevine	Improved drought tolerance, higher growth, lower leaf stomatal density, and lower photosynthesis rate	He et al., 2018
*OsNCED5, OsNCED3*	*Oryza sativa*	Rice	Enhanced drought tolerance, accelerated leaf senescence, higher ABA content	Huang et al., 2018; Huang et al., 2019
*SNAC1*			Improved drought tolerance, higher seed setting, increased stomata closure under drought	Hu et al., 2006
*ONAC022*			Enhanced drought tolerance and growth, reduced water loss and transpiration, higher proline content	Hong et al., 2016
*OsERF71*			Improved drought tolerance, increased root lignification	Lee et al., 2016
*OsTPS1*			Increased drought tolerance, higher proline and terhalose contents	Li et al., 2011b
*OsCLC1*			Enhanced drought tolerance and higher grain yield	Um et al., 2018
*AtDREB1A*	*Arabidopsis thaliana*		Increased drought tolerance, higher proline and chlorophyll, and RWC contents, higher grain yield	Ravikumar et al., 2014
*IPT*	*Agrobacterium tumefaciens*		Enhanced drought tolerance, higher grain yield, changes in the expression of genes encoding hormone-associated pathways	Peleg et al., 2011
*MnSOD*	*Pisum sativum*		Enhanced drought tolerance, reduced electrolyte leakage, higher photosynthetic rate	Wang et al., 2005
*GhABF2*	*Gossypium hirsutum*	Cotton	Improved drought and salt tolerance, increased fiber production, higher proline content and CAT activity	Liang et al., 2016b
*AREB/ABF*			Enhanced drought tolerance, induced stomatal closure, reduced transpiration and photosynthesis	Kerr et al., 2018
*StDREB2*	*Solanum tuberosum*		Improved drought tolerance, plant biomass, boll number, RWC, chlorophyll and proline contents	El-Esawi and Alayafi, 2019
*SNAC1*	*Oryza sativa*		Enhanced drought tolerance, reduced transpiration	Liu et al., 2014
*CaHB12*	*Coffea arabica*		Enhanced drought tolerance, decreased leaf abscission, lower IAA level	Basso et al., 2021
*AtHUB2*	*Arabidopsis thaliana*		Improved drought tolerance, increased boll number and boll-setting rate	Chen et al., 2019
*IPT*	*Agrobacterium tumefaciens*		Increased water-deficit tolerance, delayed senescence phenotype, higher photosynthetic capacity under drought	Kuppu et al., 2013
*betaA*	*E. coli*	Maize	Enhanced drought tolerance, higher glycine betaine content, increased grain yield	Quan et al., 2004
*AtJUB1*	*Arabidopsis thaliana*	Tomato	Increased drought tolerance, higher RWC, reduced oxidative damage and H_2_O_2_ levels	Thirumalaikumar et al., 2018
*CBF1*			Improved drought tolerance, dwarf phenotype, high proline content and CAT enzyme activity	Hsieh et al., 2002
*AtWRKY30*	*Arabidopsis thaliana*	Wheat	Improved drought and heat tolerance, enhanced growth, biomass, and gas exchange	El-Esawi et al., 2019
*AtDREB1A*			Delayed plant death upon drought stress	Pellegrineschi et al., 2004
*HaHB4*	*Helianthus annuus*		Increased drought tolerance, higher grain, spikelet, and tiller number	Gonzalez et al., 2019
*P5CS*	*Vigna aconitifolia*		Enhanced drought tolerance, higher proline and lower MDA contents, higher photosynthetic rates	Vendruscolo et al., 2007
*IPT*	*Agrobacterium tumefaciens*	Peanut	Improved drought tolerance and yield, higher photosynthetic rates and stomatal conductance	Qin et al., 2011
*AtDREB1A*	*Arabidopsis thaliana*		Improved drought tolerance; Higher transpiration efficiency, and lower stomatal conductance under normal condition	Bhatnagar-Mathur et al., 2007
*SlSIZ1*	*Solanum lycopersicum*	Tobacco	Improved drought tolerance, seed germination, lower ROS and MDA contents	Zhang et al., 2017
*P5CS*	*Saccharum officinarum*	Sugarcane	Increased drought tolerance, higher ABA and proline levels	Li et al., 2018
*NCED3*	*Arabidopsis thaliana*	Soybean	Increased drought tolerance, decreased gas exchange, reduced yield loss under drought	Molinari et al., 2020

Phytohormones are known to play a crucial role in plant response to environmental stresses including drought stress. Water deprivation alters biosynthesis of different phytohormones such as abscisic acid (ABA), auxins (IAA), gibberellins (GAs), jasmonic acid (JA), ethylene (ET), salicylic acid (SA), brassinosteroids (BRs), cytokinins (CKs). Although ABA is the major phytohormone whose production is induced by drought stress, the crosstalk between other hormones improves drought stress response in plants (Ullah et al., 2018). Two ABA-dependent and three ABA-independent regulatory pathways are involved in plant response to drought stress (Shinozaki and Yamaguchi-Shinozaki, 2007). It was shown that ABA induces the expression of most drought-related genes (e.g., *NCED*, *RD22*, *ABREs*, and *RD29*), and the up-regulation can be 40 times higher under drought stress conditions compared to under normal growth conditions (Shinozaki et al., 2003). The enzyme 9-cis-epoxycarotenoid dioxygenase (NCED) is a key enzyme that converts epoxy-carotenoid precursor to xanthonin, which is consequently converted to ABA. Overexpression of *VaNCED1* in grapevine (He et al., 2018), *OsNCED3* and *OsNCED5* in rice (Huang et al., 2018; Huang et al., 2019), and *AtNCED3* in soybean (Molinari et al., 2020) significantly improved drought tolerance in transgenic plants. The drought-responsive element (DRE)-binding proteins such as DREB1 and DREB2 are transcription factors that bind to the promoter region of dehydration-responsive genes such as *RD29A* and induce their expression in response to environmental stresses, including drought (Shinozaki and Yamaguchi-Shinozaki, 2007). The stress-inducible expression of transcriptional factor gene *DREB1A* in peanut and rice improved drought stress tolerance in transgenic plants, while its expression in wheat delayed plant death upon water deprivation (Pellegrineschi et al., 2004; Bhatnagar-Mathur et al., 2007; Ravikumar et al., 2014). Hsieh et al. (2002) showed that the ectopic expression of the *Arabidopsis* gene *CBF1 (C repeat/dehydration-responsive element binding factor 1)* in tomatoes improved plant tolerance to water deprivation, and transgenic plants accumulated more proline and showed higher CAT activity. Moreover, ectopically overexpressing *AREB/ABF* genes (coding for ABA binding factor/ABA-responsive element binding proteins) in cotton enhanced drought tolerance through stomatal regulation (Kerr et al., 2018). Transgenic cotton plants overexpressing the potato *DREB2* gene, *StDREB2*, showed improved drought tolerance with increased biomass and boll number (El-Esawi and Alayafi, 2019).

Hu et al. (2006) reported an improved drought tolerance in transgenic rice plants by overexpressing the *SNAC1* gene, which was attributed to the function of this gene in regulating stomata closure and water use efficiency. Overexpression of *SNAC1* in cotton improved salt and drought stress tolerance, enhanced the rooting system, and reduced the transpiration rate in transgenic plants (Liu et al., 2014). Furthermore, overexpression of *ONAC022* enhanced drought and salt tolerance with higher ABA biosynthesis in transgenic rice (Hong et al., 2016). Overexpression of the *JUNGBRUNNEN1* gene, *AtJUB1* (a member of the NAC family) in tomatoes increased drought tolerance associated with higher relative water content and lower H_2_O_2_ levels (Thirumalaikumar et al., 2018). Silencing the *Gossypium barbadense MYB* gene, *GbMYB5*, compromised drought tolerance in cotton, while its overexpression in tobacco enhanced drought stress response (Chen et al., 2015). It was also shown that the activities of antioxidant enzymes SOD, CAT, and POD (peroxidase) were lower and higher in silenced cotton and transgenic tobacco, respectively (Chen et al., 2015). The transcription factor gene *AtWRKY30* was overexpressed in wheat, leading to increased drought and heat tolerance (El-Esawi et al., 2019). Overexpression of *WRKY30* also induced the transcript levels of several stress-related genes such as *DREB1*, *DREB3*, *WRKY19*, and *TIP2* (El-Esawi et al., 2019). Furthermore, overexpression of the drought-responsive AP2/ERF transcription factor gene *OsERF71* enhanced drought tolerance in transgenic rice by increasing the lignification level in roots, resulting in root architecture alteration (Lee et al., 2016). Transgenic cotton plants overexpressing the *Coffea arabica HB12* gene, *CaHB12*, and *Gossypium hirsutum* bZIP transcription factor gene, *GhABF2*, independently, showed improved drought tolerance by up-regulation of genes in the ABA-dependent signaling pathway (Liang et al., 2016b; Basso et al., 2021). Recently, González et al. (2019) overexpressed the sunflower gene *HaHB4* that encodes a protein in the homeodomain-leucine zipper I family in wheat, and they showed that transgenic wheat grown in the field under drought conditions outperformed wild-type wheat.

Proline is an essential amino acid and metabolite with functions in osmotic adjustment and free radical scavenging in plants under stress conditions (Ghosh et al., 2022). Transgenic wheat and sugarcane overexpressing the *P5CS* gene showed improved drought stress tolerance associated with higher proline production (Vendruscolo et al., 2007; Li et al., 2018). As a nonreducing disaccharide, trehalose plays a vital role in plant cellular metabolism and plant response to environmental stresses (Grennan, 2007). Overexpression of the rice *trehalose-6-phosphate synthase* gene, *OsTPS1*, enhanced salt and drought tolerance in transgenic rice by increasing trehalose and proline contents (Li et al., 2011b). Previously, Jang et al. (2003) transformed rice with a gene encoding a bifunctional fusion enzyme of the TPS synthase and TPS phosphatase from *Escherichia coli*, and they observed a higher accumulation of trehalose with improved drought, salt, and cold stress tolerance. Also, overexpression of the *betaA* gene from *E. coli* in maize elevated glycine betaine levels in transgenic plants and improved drought tolerance and grain yield (Quan et al., 2004).

Overexpression of the *isopentenyl transferase* gene, *IPT* (a key gene in the biosynthesis of cytokinin), led to increased drought tolerance in transgenic peanuts (Qin et al., 2011) and rice (Peleg et al., 2011). A similar result was observed in *IPT*-transgenic cotton (Kuppu et al., 2013); however, an extended study by Zhu et al. (2018) showed that the increased drought tolerance of *IPT*-transgenic cotton depends on the timing of the occurrence of water deficit stress: if drought stress occurs after cotton starts to flower, the increased drought tolerance is lost. Transgenic rice plants overexpressing *MnSOD* showed enhanced drought tolerance (Wang et al., 2005). Recently Chen et al. (2019) showed that overexpression of *AtHUB2* that encodes a histone H2B monoubiquitination E3 ligase significantly improves boll number and boll-setting rate under drought stress conditions in transgenic cotton. Overexpression of the tomato *SlSIZ1* gene in tobacco confers increased drought tolerance, which was attributed to the accumulation of proline and SUMO conjugates (Zhang et al., 2017). Also, transgenic tobacco plants showed improved seed germination and growth while they accumulated lower amounts of ROS and malondialdehyde (Zhang et al., 2017). In rice, the root-specific expression of the *chloride channel* gene, *OsCLC1*, enhanced drought tolerance and resulted in higher grain yield in transgenic plants, whereas chloride channel mutant *osclc1* exhibited compromised drought tolerance and produced less yield than wild-type plants (Um et al., 2018).

### Transgenic approach to generate heat-tolerant crops

The average temperature of the earth is rising (Battisti and Naylor, 2009). Changes in ambient temperature accelerated by global warming affect rainfall and drought patterns across the globe, thus negatively impacting agricultural production. According to the Inter-Governmental Panel on Climatic Change (IPCC), the global average surface temperature by the end of 21^st^ century will be 0.3 - 1.7 °C under the Representative Concentraion Pathway (RCP) 2.6, 1.1 - 2.6 °C under RCP 4.5, 1.4 - 3.1 °C under RCP 6.0, and 2.6 - 4.8 °C under RCP 8.5 (IPCC, 2014). Plants’ capacity to cope with changing environments, including temperature fluctuations, varies among different species due to variations in basal and acquired thermotolerance (Grover et al., 2013). Therefore, implementation of transgenic technology is a practical solution for sustainable agriculture where transgenic crops can grow and reproduce under extreme temperatures with minimum or no damage to their cells (Mishra et al., 2017; Esmaeili et al., 2021). Here we summarize the recent advances in developing transgenic crops with improved heat tolerance (**Table 3**).

**Table 3:** Improving heat stress tolerance in transgenic crops.

Gene	Gene source	Transgenic host	Improved traits	Reference
*AtHSP101*	*Arabidopsis thaliana*	Rice	Improved heat tolerance, better growth in recovery phase, no adverse effects on growth and development	Katiyar-Agarwal et al., 2003
*mtHSP70*	*Oryza sativa*		Enhanced heat tolerance, no change in ROS production	Qi et al., 2011
*OsWRKY11*			Higher heat and drought tolerance, slow leaf-wilting	Wu et al., 2009
*BADH*	*Hordeum vulgare*		Improved heat, salt and cold tolerance, accumulation of glycinebetaine, increased root and shoot dry weight, increased numbers of tillers	Kishitani et al., 2000
*CMO*	Spinach		Increased heat and salt stress at seedling stage, higher glycinebetaine content	Shirasawa et al., 2006
*GmHSFA1*	*Glycine max*	Soybean	Enhanced thermotolerance, no abnormality in the development and growth	Zhu et al., 2006
*AsHSP70*	*Agave sisalana*	Cotton	Improved heat tolerance and boll production, higher chlorophyll, proline, and soluble sugar contents	Batcho et al., 2021
*AtHSP101*	*Arabidopsis thaliana*		Improved thermotolerance, higher pollen germination rate, increased boll set and seed numbers	Burke and Chen, 2015
*OsSIZ1*	*Oryza sativa*		Improved heat and drought tolerance, higher net photosynthesis, increased fiber yield	Mishra et al., 2017
*LeAN2*	*Lycopersicum esculentum*	Tomato	Improved heat tolerance, high photosynthetic rate, fresh weight, and antioxidant activity, lower ROS	Meng et al., 2015
*cAPX*			Increased heat tolerance, lower electrolyte leakage, higher resistance to direct sunlight in detached fruits	Wang et al., 2006
*EcDREB2A*	*Eleusine coracana*	Tobacco	Enhanced thermotolerance, seed germination, fresh and dry weight, and increased stomatal conductance	Singh et al., 2021
*OsSIZ1*	*Oryza sativa*	Creeping bentgrass	Enhanced thermotolerance, water retention and cell membrane integrity, photosynthesis, and growth	Li et al., 2013
*BADH*	*Atriplex hortensis*	Wheat	Enhanced heat and drought tolerance, higher photosynthetic rates under heat and drought stress, increased membrane stability under heat stress	Wang et al., 2010

Upon heat stress, cellular proteins lose their biological activities due to aggregation and misfolding of proteins. As a primary response to increased temperature, plants have evolved molecular chaperone and protein degradation machinery to minimize the heat-related damage in cells. The upregulation of *heat shock protein* (*HSP*) genes is an important event associated with heat stress response which results in the accumulation of HSPs as molecular chaperones to stabilize, repair, and re-fold denatured proteins, thus protecting cells against heat stress-related damages (Mittler et al., 2012). Several reports showed that overexpression of *HSP* genes in plants improves heat stress tolerance. For instance, overexpression of the mitochondrial *HSP70* gene, *mtHSP70*, and *HSP101* in rice (Katiyar-Agarwal et al., 2003; Qi et al., 2011), the *Glycine max* heat shock transcription factor gene, *GmHsfA1*, in soybean (Zhu et al., 2006) increased heat stress tolerance in transgenic plants. Transgenic cotton overexpressing *AtHSP101* (Burke and Chen, 2015) and *AsHSP70* (Batcho et al., 2021) demonstrated improved heat stress tolerance and produced more boll and seeds under high temperatures.

LeAN2 is an anthocyanin-associated R2R3-MYB transcription factor, and it was shown that overexpression of *LeAN2* in tomatoes up-regulated transcripts of several genes in the anthocyanin biosynthetic pathway and caused enhanced heat stress tolerance (Meng et al., 2015). WRKY transcription factors function as repressors and activators of gene expression, and when the *WRKY11* gene was overexpressed in rice, transgenic rice showed improved tolerance to heat and drought stresses (Wu et al., 2009). Although DREB transcription factors were first reported to be involved in plant response to drought and cold stresses, some research indicated that DREBs and HSFs could interact with each other in response to extreme heat (Grover et al., 2013). Recently Singh et al. (2021) showed that overexpression of *EcDREB2A* in tobacco improves heat stress tolerance through increasing ROS scavenging capacity in transgenic plants.

Accumulating osmolytes during the heatwave is an adaptive mechanism to protect protein’s structure in plant cells (Suprasanna et al., 2016). Overexpression of the barely peroxisomal *betaine aldehyde dehydrogenase* gene, *BADH*, in rice improved tolerance to heat, cold, and salt stresses (Kishitani et al., 2000). Transgenic wheat overexpressing *BADH* from *Atriplex hortensis* showed enhanced thermotolerance, which was attributed to a more stable membrane (Wang et al., 2010). Accumulating glycine betaine in transgenic rice overexpressing the *choline monooxygenase* gene *CMO* resulted in higher thermotolerance (Shirasawa et al., 2006). Enhanced plant biomass production and heat stress tolerance were achieved in transgenic Medicago plants overexpressing the *TPS1-TPP2* genes from yeast (Suárez et al., 2009).

Oxidative stress results from ROS accumulation in plant cells, which can be caused by many environmental stress conditions such as heat stress. Thus, utilization of genes involved in antioxidation metabolism could lead to enhanced thermotolerance in transgenic plants (Grover et al., 2013). Transgenic tomato plants overexpressing a *cytosolic peroxidase* gene, *cAPX*, increased tolerance to heat stress (Wang et al., 2006). SUMOylation is an essential post-translational modification process in plants that is also involved in abiotic stress response and the ABA-signaling pathway. Overexpression of the rice *SUMO E3 ligase* gene, *OsSIZ1*, drastically increased plant tolerance to heat and drought stresses (Li et al., 2013; Mishra et al., 2017). Transgenic cotton plants overexpressing *OsSIZ1* produced more fiber and maintained higher photosynthetic rates under heat stress conditions (Mishra et al., 2017).

## Genes pyramiding approach to improve multi-stress tolerance in crops

Plants are usually exposed to a combination of different stresses in the field; thus, plant tolerance to multiple stresses differs from their response to single stress (Zandalinas et al., 2022a and Zandalinas et al., 2022b). Therefore, more studies are required to discover the molecular mechanism of plant response to multiple stresses. In addition, crop improvement studies should focus on stress combinations that mimic field conditions (Mittler, 2006; Tian et al., 2021; Zandalinas et al., 2021a; Zandalinas et al., 2021b). Improving plant stress tolerance to complex environments such as combined drought, heat, and salt stresses is unlikely achievable if only a single gene is altered. Thus, the gene stacking strategy is a potential solution to improve crops’ tolerance to multiple stresses.

In recent years several studies have reported enhanced tolerance to multiple stresses (**Table 4**). Co-overexpression of *AVP1* and *AtNHX1* in cotton enhanced tonoplast Na^+^/H^+^ antiporter activity, leading to improved salt and drought tolerance (Shen et al., 2015). These *AVP1/AtNHX1* co-overexpressing cotton plants outperformed *AVP1*-overexpressing and *AtNHX1*-overexpressing cotton plants and produced more bolls and higher fiber yield under 200 mM NaCl. Furthermore, they produced more fiber than wild-type, *AVP1*-overexpressing, and *AtNHX1*-overexpressing cotton plants under low-irrigation and dryland conditions in the field (Shen et al., 2015). Improved salt stress tolerance was also achieved in transgenic cotton co-overexpressing *AtNHX1* and *TsVP* (Cheng et al., 2018). The *AtNHX1*/*TsVP* co-overexpressing cotton plants produced higher seed yield when grown in saline soil, which was attributed to the accumulation of Na^+^, K^+^, and Ca^2+^ in leaves and better cellular ion homeostasis in transgenic plants (Cheng et al., 2018). Transgenic tomato plants co-overexpressing *LeNHX2* and *SlSOS2* showed enhanced salt tolerance and produced more yield with enhanced fruit quality (Baghour et al., 2019). Recently, several studies employed the stacking approach to co-overexpress multiple genes in model plants, and the results are very promising. For instance, co-overexpression of *AVP1* and *PP2A-C5* in *Arabidopsis* increased tolerance to multiple stresses, including salt stress, drought stress, and phosphorous deficiencies (Sun et al., 2018). Wijewardene et al. (2020) showed that co-overexpression of the *AVP1* and the creosote *Rubisco activase* gene *RCA* in *Arabidopsis* leads to improved tolerance to drought, heat, and salt stresses. In addition, transgenic plants performed significantly better under combined drought and heat stresses, as well as under combined salt, drought, and heat stresses, and the seed yield was increased dramatically in transgenic plants. These results suggest that co-overexpression of *AVP1* and *RCA* could increase tolerance to combined drought and heat stresses and increase crop yield in light of climate changes (Wijewardene et al., 2020). In a study by Balasubramaniam et al. (2022), three genes, *AVP1*, *PP2A-C5*, and *AtCLCc*, were co-overexpressed in *Arabidopsis* to improve salt and drought tolerance, and they were able to increase the salt tolerance level up to 300 mM NaCl, a level never reached before *via* genetic engineering approach. We demonstrated that transgenic *Arabidopsis* and cotton plants co-overexpressing *AVP1* and *OsSIZ1* showed significantly improved tolerance to combined stresses such as heat with drought or drought with salt (Esmaeili et al., 2019; Esmaeili et al., 2021). In particular, in field trials under dryland conditions, transgenic cotton co-overexpressing *AVP1* and *OsSIZ1* produced 133% and 81% more fiber in two consecutive years (Esmaeili et al., 2021). These findings demonstrate that co-overexpression of multiple genes that can improve plant tolerance to abiotic stresses without imposing drawbacks on plant growth and development could be employed as an effective strategy to achieve significant tolerance towards environmental stresses.

**Table 4:** Enhanced tolerance to multiple stresses using gene pyramiding approach.

Gene	Transgenic host	Improved traits	Reference
*AVP1*/*AtNHX1*	Cotton	Improved salt and drought tolerance, increased boll and fiber yield production, higher photosynthetic rate	Shen et al., 2015
*TsVP/AtNHX1*		Enhanced salt tolerance, ion homeostasis and osmotic potential, higher RWC, carbon assimilation capacity, higher seed yield	Cheng et al., 2018
*AVP1/OsSIZ1*		Enhanced combined heat and drought and combined drought and salt tolerance, higher photosynthetic rate, RWC, increased fiber yield production	Esmaeili et al., 2021
*LeNHX2/SlSOS2*	Tomato	Enhanced salt tolerance, high leaf relative water content and water use efficiency	Baghour et al., 2019
*AtDREB1A/BcZAT12*		Enhanced drought tolerance, RWC, and yield potential, reduced electrolyte leakage (EL), hydrogen peroxide and membrane lipid peroxidation	Krishna et al., 2021
*LEA/bZIP*	Tobacco	Improved salt and osmotic stress tolerance, higher seed generation and growth rates, lower MDA and higher leaf chlorophyll contents	Qu et al., 2012
*AhBADH/SeNHX1*		Improved salt tolerance, higher betaine and Na^+^ levels, greater biomass, increased osmotic pressure	Zhou et al., 2008
*OsbZIP46CA1/SAPK6*	Rice	Improved drought, heat and cold tolerance, higher yield, biomass, spikelet number, and grain number	Chang et al., 2017
*OsGS1;1/OsGS2*		Increased osmotic, salt, and drought tolerance, higher grain filling, increased poline and reduced MDA contents	James et al., 2018
*NCED3/ABAR/CBF3/LOS5/ICE1*	Rapeseed	Enhanced heat, salinity, osmotic stress, and cold tolerance, greater yield, biomass, spikelet number, and grain number	Wang et al., 2018
*PaSOD/RaAPX*	Potato	Increased salt tolerance, starch accumulation, enhanced growth, reduced ROS accumulation	Shafi et al., 2017
*codA/SOD/APX*		Enhanced salt and drought tolerance, higher glycine betaine level, lower levels of H_2_O_2_	Ahmad et al., 2010
*APX/SOD*		Higher heat and oxidative stress tolerance	Tang et al., 2006
*betaA/TsVP*	Maize	Enhanced drought tolerance, better water uptake, improved osmotic adjustment	Wei et al., 2011

Gene pyramiding of the *Arabidopsis* gene *AtDREB1A* with the Brassica Zinc finger transcription factor gene *BcZAT12* in tomato improved drought tolerance and fruit production (Krishna et al., 2021). These transgenic tomato plants also showed an elevated water use efficiency with higher proline content, while electrolyte leakage, hydrogen peroxide level, and membrane lipid peroxidation were significantly reduced (Krishna et al., 2021). Co-overexpression of the *late embryogenesis abundant protein* gene *LEA* and the *basic leucine zipper transcriptional factor* gene *bZIP* in tobacco enhanced tolerance to salt and osmotic stresses, resulting in increased seed production (Qu et al., 2012). Previously, co-overexpression of the constitutively active form of a bZIP transcription factor gene *OsbZIP46CA1* and a protein kinase gene *SAPK6* in rice enhanced tolerance to drought, heat, and cold stresses (Chang et al., 2017). The gene pyramiding approach was also employed to generate transgenic rapeseed (*Brassica napus*) with novel traits (Wang et al., 2018). Five stress-related genes, *NCED3* (*nine-cis-epoxycarotenoid dioxygenase 3*), *ABAR* (*ABA receptor*, magnesium-chelatase subunit chlH), *CBF3* (*c-repeat binding factor 3*), *LOS5* (*molybdenum cofactor sulfurase*), and *ICE1* (*interactor of little elongation complex ELL subunit 1*) were simultaneously expressed in rapeseed plants, which led to improved tolerance to multiple stresses such as heat, salinity, osmotic stress, and cold (Wang et al., 2018).

Glutamine synthetase (GS) is known for its function in the nitrogen metabolism, in which its activity and expression are affected by different abiotic stresses (Ji et al., 2019). Recently, James et al. (2018) developed transgenic rice plants that co-overexpress the rice cytosolic *GS1* and chloroplastic *GS2* genes, *OsGS1;1* and *OsGS2*, and they observed improved osmotic, salt, and drought tolerance in transgenic plants. In addition, the grain filling rate in transgenic rice was dramatically higher than that in control plants under salinity and drought stresses, which was associated with higher proline and lower MDA contents (James et al., 2018). In transgenic potatoes, enhanced heat stress tolerance and increased antioxidation capacity were achieved by co-overexpressing a Cu/Zn *superoxide dismutase* gene *SOD* and an *ascorbate peroxidase* gene *APX* in the chloroplast (Tang et al., 2006). Co-overexpression of ROS scavenging enzymes genes *PaSOD* and *RaAPX* in potato increased starch accumulation and enhanced growth under salt stress conditions, while it reduced accumulation of ROS because of the higher APX and SOD activities in transgenic plants (Shafi et al., 2017). Transgenic potato plants co-overexpressing the genes *codA*, *SOD*, and *APX* showed improved salt stress tolerance (Ahmad et al., 2010).

Co-overexpression of the Atriplex betaine synthesis gene *BADH* with the Salicornia *NHX1* gene *SeNHX1* in tobacco resulted in a higher accumulation of betaine and Na^+^ in transgenic lines under salinity stress, and these *BADH/SeNHX1* co-overexpressing plants produced more biomass and maintained increased osmotic pressure (Zhou et al., 2008). Wei et al. (2011) employed a similar approach to simultaneously overexpress *betaA* from *Escherichia coli* and *TsVP* from *Thellungiella halophila* in maize. Their study showed that *betaA/TsVP* co-overexpressing maize lines performed better than wild-type plants under drought stress conditions, with an enhanced osmotic adjustment that facilitates water uptake by roots (Wei et al., 2011).

Although the gene stacking approach is an effective and promising strategy to improve plant tolerance to abiotic stresses, there might be some drawbacks associated with this approach. Overexpression of single target genes such as those encoding specific enzymes or transporters could improve plant performance against individual stresses in most cases, or two related stresses such as drought and salt stresses, while manipulating the upstream regulatory genes such as transcriptional factor genes could result in enhanced tolerance against multiple abiotic stresses. However, due to the complex cross-talks among regulatory and metabolic pathways in plants, altering upstream genes might also lead to undesired agronomical traits, including growth retardation. Therefore, as mentioned above, an appropriate combination of genes to ensure the synergistic interactions of the genes is critical. Since promoters can significantly affect the outcomes from a transgenic alteration, selecting a proper combination of promoters for the genes is also crucial.

## Application of CRISPR/Cas9 technology to engineer abiotic stress tolerance in plants

Recently, clustered regularly interspaced short palindromic repeats-associated protein 9 (CRISPR/Cas9) technique has emerged as a promising gene editing technology with a great potential for precise genetic modification, aiming to improve abiotic stress tolerance in plants (Li et al., 2022). Unlike zinc-finger nucleases (ZFNs) and transcription activator-like endonucleases (TALENs), the CRISPR/Cas9 endonuclease system is a very fast, accurate, and highly efficient genome editing tool to introduce desirable traits in plants for crop improvement (Rao and Wang, 2021). Genome editing using the CRISPR/Cas9 knock-out system has produced several crops with enhanced environmental stress tolerance. The simultaneous knock-out of three abscisic acid receptor genes, *OsPYL1*, *OsPYL4*, and *OsPYL6*, improved heat stress tolerance in rice, which leads to increased yield production (Miao et al., 2018). In plants, mitogen-activated protein kinases (MAPKs) are highly conserved serine and threonine protein kinases that are involved in plant development, hormone regulation, and response to abiotic stresses. In a study by Yu et al. (2019), the *SlMAPK3* gene, a member of the MAPK family in tomatoes, was knocked out using the CRISPR/Cas9 technique. The *slmapk3* mutants showed an improved heat stress tolerance, and the transcripts of several *HSP* and *HSF* genes were upregulated under stress conditions (Yu et al., 2019). Recently, Zeng et al. (2020) employed CRISPR/Cas9 technology to generate three knock-out mutants in rice by editing *OsPIN5b*, *GS3*, and *OsMYB30*, and they showed that the *osmyb30* (a cold-responsive R2R3-type *MYB* gene) mutants displayed improved cold tolerance, while *ospin5b* (a gene involved in balance and transport of auxin) and *gs3* mutants showed increased panicle length and enlarged grain size, respectively. In addition, the simultaneous knockout of all three genes resulted in enhanced cold tolerance and higher yield production compared to WT plants (Zeng et al., 2020). The auxin response factor (ARF) regulates the auxin-responsive genes in plants. It was shown that the knockouts of *SlARF4* in tomato improved tolerance to drought stress and increased rehydration ability *via* upregulation of ABA signaling pathway genes such as *SlABI5/ABF* and *SCL3* (Chen et al., 2021). In addition, *arf4* mutants showed a higher level of antioxidant enzyme activities compared to WT plants, and no significant decrease in photosynthetic efficiency was observed (Chen et al., 2021). Furthermore, the antisense down-regulation of *SlARF4* in tomato plants enhanced salinity and osmotic stress tolerance. In addition, plants showed higher levels of soluble sugars and chlorophyll contents and enhanced root growth under stress conditions (Bouzroud et al., 2020). The *ARF4* antisense plants also maintained higher relative water content in leaves and ABA content under both normal and stress conditions, which was attributed to the upregulation of several ABA biosynthesis genes (Bouzroud et al., 2020). The *ARGOS8* is considered a negative regulator of ethylene responses in maize plants, but its mRNA level is relatively low in maize. In an effort by Shi et al. (2017), novel *ARGOS8* variants harboring native maize GOS2 promoter were generated using the CRISPR-Cas9 technology. The results showed that the *ARGOS8* transcripts level was elevated in *ARGOS8* variants. In addition, the *ARGOS8* variants produced significantly higher grain yield than WT plants when grown under drought stress conditions in the field (Shi et al., 2017). Furthermore, the targeted mutagenesis of the Rice Enhanced Response to *ABA1* gene, *OsERA1*, improved ABA response and drought stress tolerance in osera1 rice mutants (Ogata et al., 2020).

The CRISPR/Cas9 system was also employed to enhance salt stress tolerance in rice at the seedling stage *via* targeted mutagenesis of the transcription factor gene *OsRR22*, and no significant differences in agronomy traits were observed between the *osrr22* mutant and WT plants (Zhang et al., 2019). Tran et al. (2021) showed that the negative-response domain in *SlHyPRP1* was precisely removed in tomato hybrid plants using the CRISPR/Cas9 method. The tomato *hybrid proline-rich protein 1*, *HyPRP1*, is considered a negative regulator of salt stress; however, its elimination in tomato plants resulted in an increased salt stress tolerance at both germination and vegetation stages (Tran et al., 2021). The ABA-induced transcription repressors (AITRs) are members of the transcription factor family involved in the ABA signaling pathway. It was reported that the targeted mutation of *GmAITR* in soybean *via* the CRISPR/Cas9 gene editing strategy leads to improved salinity tolerance in mutants grown in the field (Wang et al., 2021). In a study by Lou et al. (2017), the functional properties of osmotic stress/ABA–activated protein kinase 2 (SAPK2) in rice was investigated. The *sapk2* mutants generated *via* CRISPR/Cas 9 showed ABA-insensitive phenotypes during germination and reduced tolerance to drought stress, indicating that SAPK2 is involved in drought stress response in rice (Lou et al., 2017).

The *nonexpressor of pathogenesis-related gene 1* (*NPR1*) is a salicylic acid receptor, and its function in plant response to pathogens has been well documented (Wu et al., 2012). However, there is a poor understanding of *NPR1*’s role in regulating plant response to abiotic stresses (Li et al., 2019). Recently, CRISPR/Cas9 technique was employed to generate mutations in the *NPR1* gene in tomato plants. The *slnpr1* mutants showed reduced drought stress tolerance with a significant decrease in the transcript level of several drought-related genes, including *SlGST*, *SlDHN*, and *SlDREB* (Li et al., 2019). In rice, the leaf morphology is a critical agronomical trait where rolled leaves show reduced water loss and improved drought tolerance compared to semi-rolled leaves. CRISPR/Cas9-based mutagenesis of *semi-rolled leaf1* and *leaf2* genes, *SRL1* and *SRL2*, in rice resulted in rolled leaf mutants with enhanced drought stress tolerance and grain filling percentage compared to WT plants (Liao et al., 2019).

The CRISPR from *Prevotella* and *Francisella 1* (Cpf1) is a single RNA-guided endonuclease with several advantages that sets it apart from the Cas9 system. Unlike CRISPR/Cas9 system, the CRISPR-Cpf1 results in higher transformation efficiency because it does not require large constructs to express multiple sgRNA cassettes (Wang et al., 2017). With high efficiency, engineered CRISPR-Cpf1 was recently used for multiplex gene editing in rice plants. The *Francisella novicida* Cpf1 (FnCpf1) was used to edit four members of rice *Related to receptor-like kinases* (*OsRLK*s) and *Lachnospiraceae bacterium* ND2006 Cpf1 (LbCpf1) was employed to edit four members of the rice *Bentazon‐sensitive‐lethal* (*OsBEL*s). The analysis of T0 transgenic rice plants showed successful multiplex gene editing, indicating that engineered CRISPR-Cpf1 could be utilized as a powerful tool to target multiple members of a gene family (Wang et al., 2017). Overall, CRISPR/Cas9 gene editing technique involves simple designing and cloning methods that plant biotechnologists could use as an alternative tool for crop improvement. This technique has been employed in several different crop species, such as rice, wheat, maize, tomato, and soybean, to improve their yield and/or their response to biotic and abiotic stresses. Although it is a highly efficient and fast tool, CRISPR/Cas9 is relatively new and has been modified in many functional studies, further improvements are likely needed (Jaganathan et al., 2018).

## Conclusion and future road map

This review briefly summarizes the recent advances in developing transgenic crops to combat major abiotic stresses, including heat, drought, and salinity. Despite the progress in improving abiotic stress tolerance in crops, the assessment of stress tolerance in transgenic plants has been largely carried out in the laboratory and/or greenhouse under controlled conditions where plants are not exposed to other stress conditions related to the field. In many cases, laboratory stress tolerance assays use nutrient-rich media containing sucrose, which shows no relationship with field conditions. Therefore, the abiotic stress tolerance of transgenic crops must be evaluated in the field, and more importantly, the yield potentials of these transgenic crops should be assessed. Evaluating the performance of transgenic plants in the field is challenging due to the complexity and variability of stresses in the field (Esmaeili et al., 2021). The concurrence of multiple stresses and the potential for interactions with other field factors such as soil fertility, light intensity, soil pH, presence of different salts and toxic elements, temperature, humidity, mechanical stress from strong wind, and transpiration and water loss make plant stress evaluation in the field very difficult (Yamaguchi and Blumwald, 2005). Therefore, developing multi-stress tolerant crops, particularly those that could quickly adapt to the changing environments, should be prioritized.

Furthermore, most studies on developing abiotic stress-tolerant plants have been carried out on model plants such as *Arabidopsis*, tobacco, and rice. Thus, it is critical to generate transgenic crops using the knowledge we learned from studying model plants (Mittler, 2006). Although rice is an important crop in addition to being a model plant for monocots, transforming other monocots has been time-consuming, expensive, and challenging (Yamaguchi and Blumwald, 2005). Despite the tremendous progress in improving crops through the biotechnology approach, a better understanding of plant stress response and the tolerance mechanism is urgently needed (Zandalinas et al., 2022a; Zandalinas et al., 2022b). Indeed, a more precise and comprehensive understanding of the underlying mechanisms of plant response to stresses will help us to design climate-resilient crops for the future (Dita et al., 2006; Tian et al., 2021; Rivero et al., 2022). The emergence of several functional tools over the past decades has assisted researchers in unraveling the underlying mechanisms of stress tolerance in plants. For instance, marker-assisted selection (MAS) enabled researchers to construct associated gene maps and identify quantitative trait loci (QTL) responsible for improved stress tolerance. The genome-wide association study in crops covering the whole genome could detect major QTLs in crops responsible for enhancing abiotic stress tolerance (Abdelraheem et al., 2019). Other emerging technologies, including gene editing tools such as CRISPR/Cas9 and Transcription Activator-Like Effector Nucleases (TALEN), are examples of new technologies that give promise to the future of crop biotechnology. The advantage of CRISPR/Cas9 in genome editing involves alteration of a few nucleotides in the original DNA of an organism without introduction of a large foreign DNA fragment, which could help the acceptance of genetically modified organisms (GMO) in the rest of the world as only minimal changes are made in the crop genomes.

Crops lack many beneficial traits of their wild-type relative species, such as disease resistance and abiotic stress tolerance, due to extensive breeding and domestication that occurred over millennia (Zsögön et al., 2018). Recent studies on the domestication of crops show that only a limited number of genes have been altered through this process, and in fact, some of these genes are highly conserved among different species. This collective evidence has driven an increasing interest in *de novo* domestication (dnD) and re-domestication of crops (Fernie and Yan, 2019). Recent advances in gene editing technology and the *de novo* domestication approach have opened promising avenues to generating crops by altering domestication-related genes in wild species. The *de novo* domestication platform uses CRISPR–Cas9 targeted genome editing technology to manipulate crops’ wild relatives by targeting specific genes linked to stress tolerance and/or nutritional quality (Zsögön et al., 2017). Although there are not many reports on using *de novo* domestication, Zsögön et al. (2017) examined the domestication of the world’s six main crops, including maize, rice, and wheat. They suggested that the key monogenic traits could be introduced into wild relatives of crops *via* gene editing technique (Zsögön et al., 2017). Recently, the wild relative of the present-day tomato crop, wild *Solanum pimpinellifolium* was domesticated *de novo via* editing six loci. The engineered lines produced fruits threefold and tenfold larger in size and number, respectively, compared with the wild parent (Zsögön et al., 2018). Therefore, the genetic diversity of wild species can be utilized in molecular breeding for the domestication of wild plants by targeting agronomically valuable traits such as improved stress tolerance, nutritional features, and enhanced yields (Gasparini et al., 2021).

On the other hand, the most globally consumed crops, including rice, maize, wheat, soybean, sugarcane, potato, and tomato, are mainly exotic species. Recent reports demonstrate that re-domestication of these plant species through gene editing or selective breeding could be an alternative approach to growing exotic species (Fernie and Yan, 2019). It has been proved that the gene editing strategy is a valuable and reliable technique to improve target traits accurately and rapidly in plant species. Recent progress in knowledge-driven re-domestication and *de novo* domestication of crops opens up promising doors to achieve improved crop tolerance and yield production.

Reports suggest that “gene discovery” is an important limiting factor in the genetic engineering of plants. Whole-genome sequencing, along with omics technology (e.g., genomics, proteomics, and metabolomics), will likely lead to identifying different genes expressed under stress conditions. Such novel genes could be used as potential candidates to enhance plant stress tolerance. Identification of stress-related metabolites in crops can be essential in improving plant stress tolerance. Because overexpression of single genes in crops leads to a limited increase in stress tolerance, the gene pyramiding approach in which several functionally related genes are simultaneously overexpressed appears to be a more logical strategy. Nevertheless, recent progress in improving plant tolerance against a combination of abiotic stresses *via* multi-gene assembly raises a solid hope to tackle the negative impacts of the abiotic stresses on agricultural production. Thus, the main goal in attaining sustainable agriculture is to gather and implement the knowledge we gained to develop crops that can grow and reproduce successfully in a complex environment.

## Author contributions

NE wrote the first draft, GS and HZ participated in discussion and revision of the manuscript. All authors approved the final manuscript.

## Funding

This work was partly supported by grants to Guoxin Shen from the Key Technologies R & D Program for Crop Breeding of Zhejiang Province (2021C02072-5) and the Natural Science Foundation of China (31402140).

## Conflict of interest

The authors declare that the research was conducted in the absence of any commercial or financial relationships that could be construed as a potential conflict of interest.

## Publisher’s note

All claims expressed in this article are solely those of the authors and do not necessarily represent those of their affiliated organizations, or those of the publisher, the editors and the reviewers. Any product that may be evaluated in this article, or claim that may be made by its manufacturer, is not guaranteed or endorsed by the publisher.
